# Developing a Framework for Population Health in Interprofessional Training: An Interprofessional Education Module

**DOI:** 10.3389/fpubh.2019.00058

**Published:** 2019-03-21

**Authors:** Olivia S. Anderson, Ella August, Phoebe K. Goldberg, Emily Youatt, Angela J. Beck

**Affiliations:** ^1^Department of Nutritional Sciences, School of Public Health, University of Michigan, Ann Arbor, MI, United States; ^2^Department of Epidemiology, School of Public Health, University of Michigan, Ann Arbor, MI, United States; ^3^Department of Health Behavior and Health Education, School of Public Health, University of Michigan, Ann Arbor, MI, United States

**Keywords:** interprofessional education, interprofessional learning, interdisciplinary, public health, professional skills, teamwork, population health, undergraduate education

## Abstract

Interprofessional education (IPE) is based on the concept that health professional students are best trained on the skills, knowledge, and attitudes that promote population health when they learn with and about others from diverse health science fields. Previously, IPE has focused almost exclusively on the clinical context. This study piloted and evaluated an IPE learning experience that emphasizes population health in a sample of public health undergraduate students. We hypothesized that students who completed the 2-hour online asynchronous module would better understand the value of public health's role in interprofessional teams, the benefit of interprofessional teamwork in improving health outcomes, and the value of collaborative learning with other interprofessional students. Students engaged in pre- and post-training assessments and individual reflections throughout the module. Sixty-seven undergraduate public health students completed the module and assessments. After completion, a greater proportion strongly agreed that students from different health science disciplines should be educated in the same setting to form collaborative relationships with one another (19 vs. 39% before and after completion, respectively). A greater proportion also strongly agreed that care delivered by an interprofessional team would benefit the health outcomes of a patient/client after the training (60 vs. 75% before and after, respectively). Mean scores describing how strongly students agreed with the above two statements significantly increased post-training. A greater proportion of students strongly agreed that incorporating the public health discipline as part of an interprofessional team is crucial to address the social determinants of health for individual health outcomes after taking the training (40 vs. 55% before and after, respectively). There was little change in attitudes about the importance of incorporating public health as part of an interprofessional team to address social determinants of health for population health outcomes, which were strongly positive before the training. Most students reported being satisfied with the module presentation and felt their understanding of interprofessional practice improved. This training may be useful for students from all health disciplines to recognize the benefits of engaging with and learning from public health students and to recognize the important role of public health in interprofessional practices.

## Introduction

The premise for interprofessional education (IPE) is that students from different health science backgrounds actively engage together early in their training to develop skills necessary to collaborate successfully (1, 2). Although IPE is often included within graduate-level health professional programs, mastering interprofessional competencies is important to implement early in training. This is especially the case for the health science fields, like public health, that have formalized, accredited undergraduate programs with objectives that mirror the IPE competencies. Public health education emphasizes areas like communication, teamwork, values, and health promotion (3). IPE curricular materials tailored for undergraduate education are designed to improve knowledge about interprofessional collaboration and care and to shape attitudes, behaviors, and values that support this practice in graduate training and professional practice (4–7). As the student develops professionally, these skills are poised to support all aspects of the “quadruple aim” of health care leading to (1) equitable access to care; (2) improvement in the quality of patient care; (3) enhancement of practitioner experience; and (4) improvements in population health (8).

Ample opportunities have been developed for health professional learners to engage in IPE, but most take the form of a clinically-focused case study or clinical simulated experience. The narrow emphasis on clinical learning environments prompted a movement for the Interprofessional Education Collaborative to expand their model of interprofessional care to include population health (8). Including population health enables a framework for clinical care providers, public health practitioners, and professionals from other non-clinical health fields to collaborate effectively and creatively together across disciplines to advance health at the population level. This expanded IPE model opens up the opportunity to develop interprofessional learning environments that take into careful consideration the non-clinical team members from fields like public health and their specific role in the health care process (9).

In addition to the lack of undergraduate- and nonclinical-based IPE learning tools, IPE is often challenging to integrate into the tightly-prescribed curricula of public health programs that comply with accreditation standards (10). Online learning provides a dynamic way to offer innovative pedagogy related to IPE beyond the traditional, in-person, on-site classroom or laboratory setting. Online learning platforms are accessible, convenient, and cost-effective. These platforms offer active and engaging learning environments for diverse targeted audiences, including undergraduate health science students.

Herein, we describe a pilot and evaluation of an online, asynchronous IPE module, tested with public health undergraduate students. This module is timely, with conferred undergraduate public health degrees increasing by 11% from 2003 to 2016 (3). The module provides a didactic overview of what IPE is and how public health plays an integral role in an interprofessional team. Its availability in a portable, online format provides a practical opportunity to implement it beyond this pilot with undergraduate public health students and to engage other disciplines to learn about public health as a key component of the framework for interprofessional collaboration and care. We referenced the interprofessional learning continuum model for this study, which shows that IPE activities comprise a smaller component of foundational education initiatives early in the learning continuum, as students are being introduced to their profession (11, 12). This intervention is intended to be an initial exposure to IPE, which students build on through graduate work or on-the-job training in future years. We hypothesized that students who completed the two-hour online asynchronous module would better understand the value of public health's role in interprofessional teams, the benefit of interprofessional teamwork in improving health outcomes, and the value of collaborative learning with interprofessional students.

## Materials and Methods

### Module Design

The module, *Interprofessional Practice for Population Health*, was developed in 2018 by the Michigan Public Health Training Center at the University of Michigan School of Public Health to address a training need among students and public health professionals. Learning objectives for the module are identified in Table 1.

**Table 1:** Learning objectives of *Interprofessional Practice for Population Health* module.

**Module learning objective**	**Content addressing learning objective**
Define the four core competencies of interprofessional education	Part 1—This section defines each of the four IPEC competencies (8), discusses the importance of each skill, and provides examples of what each looks like in practice.
Define population health from a public health perspective	Part 2—Content describes how the term “population health” is used differently in medicine vs. public health (13), and provides the Kindig and Stoddart definition of population health as “the health outcomes of a group of individuals, including the distribution of such outcomes within the group” (14).
Define prevention from a public health perspective	Part 2—The three levels of prevention—primary, secondary, and tertiary—are defined, in part to illustrate the difference in focus between public health and health care.
Recognize real-world applications of interprofessional practice	Parts 3–5—Each of these sections presents a different real-world example as described in the narrative.

The IPE module is a self-paced online training that takes approximately two hours to complete and is divided into five parts. The first part was developed by a University of Michigan faculty with expertise in IPE. This section provides an overview of IPE—including the four Interprofessional Education Collaborative competency domains of values/ethics, roles/responsibilities, interprofessional communication, and teams/teamwork (8). The second part was created by the Michigan Public Health Training Center's program manager and establishes the importance of interprofessional partnerships for effective population health practice and outcomes.

The remaining three parts of the module feature three real-world examples of interprofessional collaboration. Throughout the three examples, practitioners discuss benefits, challenges, lessons learned, and recommendations for working across disciplines in order to influence community health. In Part 3, a tribal court judge and the tribal court's Healing to Wellness program manager describe how law enforcement and numerous types of health professionals work together to address opioid misuse in their community with a culturally relevant alternative sentencing program that supports participants with health and other services. In Part 4, an emergency room nurse and a peer support specialist explain the emerging recognition of human trafficking as a population health concern and several initiatives of Michigan's human trafficking task forces, which are composed of members from health, law, policy, and other fields as well as survivors themselves. Lastly, Part 5 features a public health professional and a land policy educator who explain the frameworks of Health in All Policies and Complete Streets as examples of cross-sector, team-based approaches for advancing healthier built environments. The three topics of opioid misuse, human trafficking, and Health in All Policies were selected as focus areas given their prominence in national conversations about population health outcomes and strategies, and examples in which collaboration across sectors is needed for system-level change.

### Modality and Development

The module was created using Articulate Storyline 3, a software program used for online learning development (15). Production of the module took place across ~ 9 months from conception to launch. The Michigan Public Health Training Center staff led the identification, recruitment, and coordination of presenters during this process. The Center's program manager worked with presenters face-to-face and by phone to shape the content in alignment with the module's learning objectives. The program manager and instructional designer then hired professional videographers to document the three practice-based vignettes. The instructional designer compiled the presentations and recordings into a single online training file, with visuals designed to have a consistent look and feel.

The first two parts of the module feature presentation slides with audio voiceover. The three remaining parts include video narrative from the practice-based professionals as well as presentation slides. Each part lasts approximately between 10 and 30 min. Brief opportunities for interaction are dispersed throughout the module in the form of multiple-choice and reflective open-ended questions. For example, after receiving information about the benefits of interprofessional and cross-sector partnerships to public health practice, students are asked to write a response about how public health practitioners can work with other professions to maximize their impact in one of the 10 Essential Services of Public Health. Other interactive questions throughout the module focus on connecting the material with the concept of teamwork.

The training is hosted through the Michigan Public Health Training Center's Canvas Catalog learning management system. The final version of the training is publicly available at www.mitrainingcenter.org. The present study used a pilot version of the training module.

## Training Implementation

### Participants

The module pilot was conducted with a cohort of 92 senior undergraduate students earning either a Bachelor of Arts in Community and Global Public Health or a Bachelor of Science in Public Health Sciences at the University of Michigan. In the Fall 2018 semester, *Interprofessional Practice for Population Health* was offered as an extra credit activity for the entire cohort of students through an academic course focused on public health practice and professional development. Incentives to participate included a certificate of completion and a seasonal snack for the class if 80% or more completed the module requirements.

Students in the academic course were invited to enroll in the training through a given link and to complete the online module requirements of a pre-survey, participation, post-survey (evaluation), and post-quiz (passing at 80%+), if interested. These are the same requirements for professionals taking the final version of the module, although the survey questions in this study were modified for the pilot undergraduate audience, as described below.

### Evaluation Methods

Evaluation tools drew upon several existing resources. The pre- and post-surveys included one item (Q1a) adapted from the validated Student Perceptions of Physician-Pharmacist Interprofessional Clinical Education—Revised 2 (SPICE-R2) questionnaire (16). Staff from the Michigan Public Health Training Center and a University of Michigan Public Health faculty member developed three additional items to examine students' attitudes about the effect of interprofessional teamwork on patient health outcomes and the importance of public health in interprofessional teams for addressing the social determinants of health for individual and population health outcomes. Response options were a 6-point Likert scale ranging from *strongly disagree* to *strongly agree*. Post-survey questions were designed to assess participants' behavioral intentions to apply what was learned and general satisfaction with the module. The post-module quiz included questions designed to assess achievement of the learning objectives. See the Supplementary Materials for the specific quiz questions.

The pilot module was available to students in the academic course for 16 days, after extending the original 9-day invitation time by seven days to accommodate more student participation. Students received several reminders from their course instructor throughout that time. Students were removed from analysis if their data could not be matched across all three sources (pre-survey, post-survey, post-quiz).

### Statistical Analysis

Frequencies were calculated for pre- and post-survey data. In addition, a paired *t*-test was used to compare mean differences in pre- and post-survey data for the items examining student attitudes. The analyses were conducted using Microsoft Excel and IBM SPSS software (version 24, IBM Corp, Armonk, NY). This study was self-determined as Not Regulated through the University of Michigan Health Sciences and Behavioral Sciences Institutional Review Board (HUM00153475).

## Results

A total of 88 public health undergraduate students enrolled in the module within the timeframe in which it was available. Of the students who enrolled, 67 (73% response rate from the 92 invited) were included in the data analysis. Over half of the students (55%; *n* = 37) reported spending < 2 hour to complete the module, 40% (*n* = 27) spent 2 hour and 5% (*n* = 3) spent two and a half hours or more to complete it.

**Figure 1:**
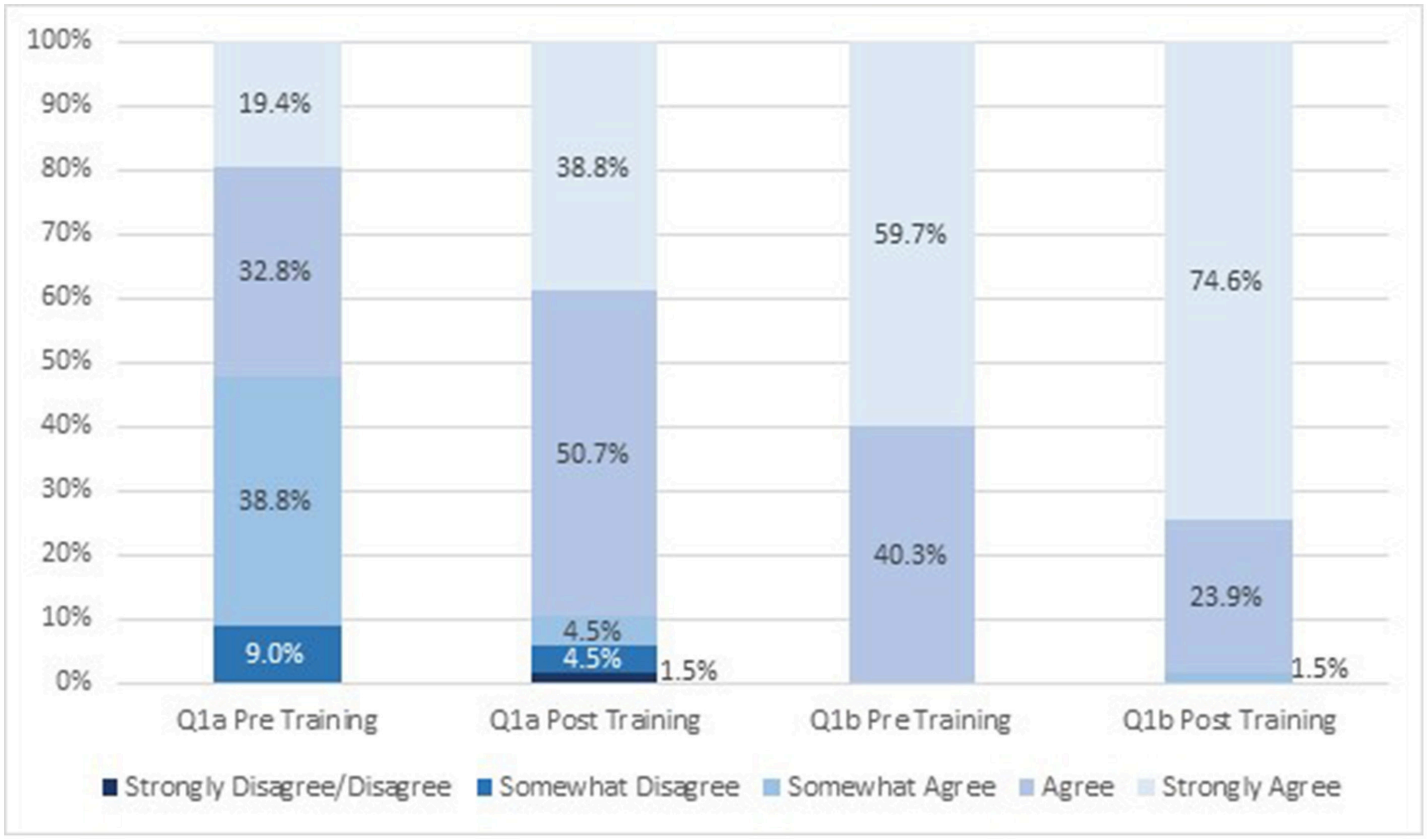
Attitudes about interprofessional education before and after engaging in the module (*n* = 67). Q1a: Students from different health science disciplines should be educated in the same setting to establish collaborative relationships with one another. Q1b: Care delivered by an interprofessional team will benefit the health outcomes of a patient/client.

**Figure 2:**
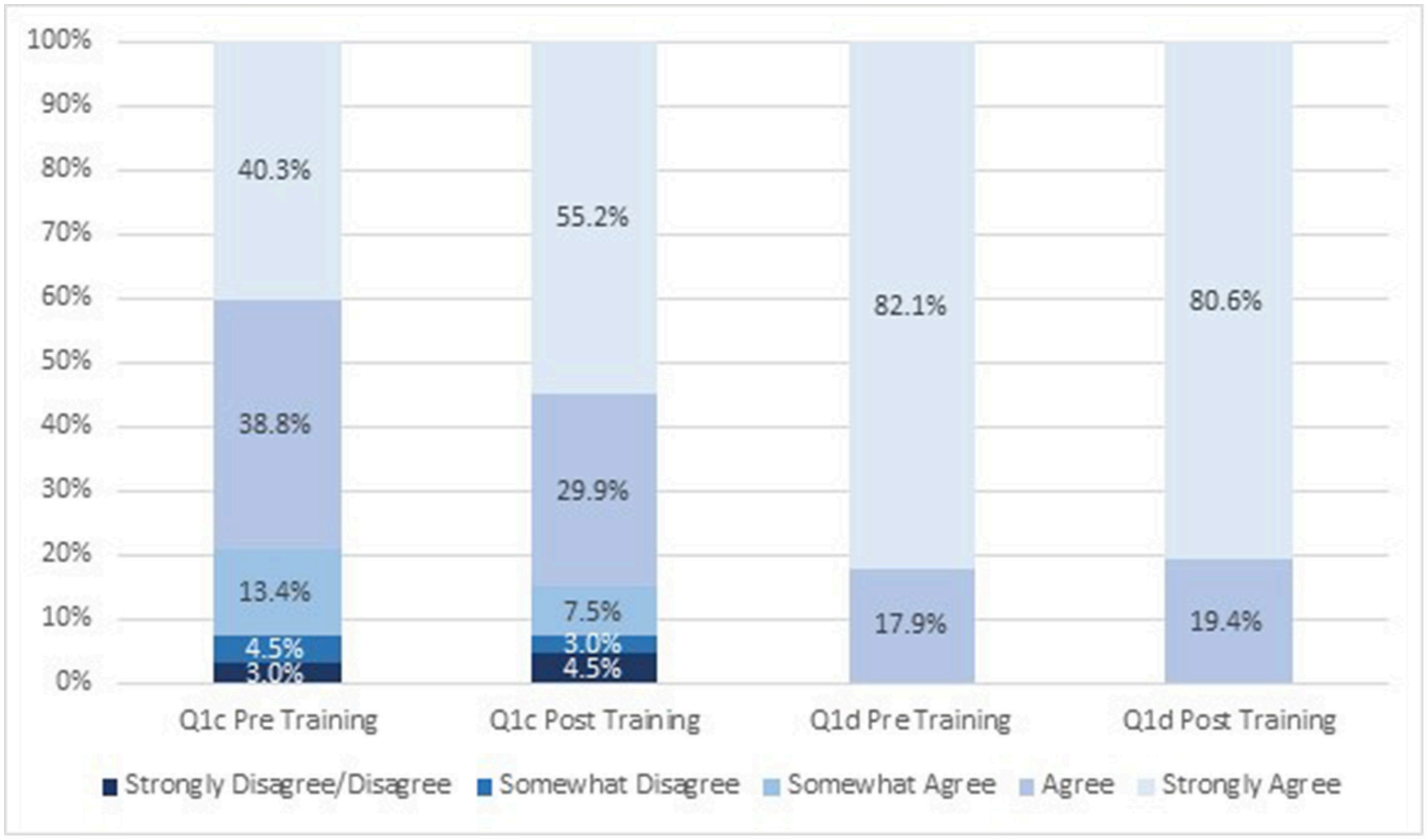
Attitudes about interprofessional education before and after engaging in the module (*n* = 67). Q1c: Incorporating public health as part of an interprofessional team is crucial to address the social determinants of health for individual health outcomes. Q1d: Incorporating public health as part of an interprofessional team is crucial to address the social determinants of health for population health outcomes.

Student attitudes about IPE changed after viewing the module (Figures 1, 2). After participating in the module, a greater proportion of respondents strongly agreed (19 vs. 39% before and after participating, respectively) that students from different health science disciplines should be educated in the same setting to establish collaborative relationships with one another (Figure 1). After completing the module, a greater proportion of students strongly agreed that care delivered by an interprofessional team would benefit the health outcomes of a patient/client (60 vs. 75% before and after completing, respectively; Figure 1). Similarly, a greater proportion of students strongly agreed that incorporating the public health discipline as part of an interprofessional team is crucial to address the social determinants of health for individual health outcomes after taking the training (40 vs. 55% before and after taking, respectively; Figure 2). There was little change in attitudes that incorporating public health as part of an interprofessional team is crucial to address the social determinants of health for population health outcomes (18 vs. 19% agreed before and after completing the module, respectively, and 82 vs. 81% strongly agreed before and after completing the module, respectively; Figure 2). The mean scores for the first two survey items significantly increased post-training (Table 2). There was no change in attitudes around the importance of incorporating public health as part of an interprofessional team to address social determinants of health for population health outcomes, which were strongly positive before the training.

**Table 2:** Mean differences in attitudes toward IPE pre- and post-training (*n* = 67).

**Attitudes toward IPE**	**Mean pre score**	**Mean post score**	**95% CI**	***t***	***p*-value**
Q1a. Students from different health science disciplines should be educated in the same setting to establish collaborative relationships with one another.	4.64	5.21	−0.78, −0.35	−5.3	**<0.001**
Q1b: Care delivered by an interprofessional team will benefit the health outcomes of a patient/client.	5.52	5.73	−0.34, −0.08	−3.18	**0.002**
Q1c: Incorporating public health as part of an interprofessional team is crucial to address the social determinants of health for individual health outcomes	5.09	5.25	−0.40, 0.07	−1.42	0.161
Q1d: Incorporating public health as part of an interprofessional team is crucial to address the social determinants of health for population health outcomes.	5.81	5.81	−0.09, 0.09	0.0	1.0

*Bold values are considered statistically significant*.

Most students reported being satisfied with the presentation of the module and felt they benefited from it. Sixty-seven percent reported that the presenters in the video were effective and 84% indicated that the information was presented in a clear and understandable way (Table 3). Three-quarters of participants reported that the module improved their understanding of interprofessional practice and 72% reported that they planned to apply the information from the module to their continuing education. Finally, 66% of students were satisfied with the module overall and 50% would recommend it to another student.

**Table 3:** Public Health undergraduate student evaluations of the IPE module (*n* = 67).

	**Strongly disagree % (N)**	**Disagree % (N)**	**Somewhat disagree % (N)**	**Somewhat agree % (N)**	**Agree % (N)**	**Strongly agree % (N)**
The presenters were effective	0	1.5 (1)	7.5 (5)	23.9 (16)	49.3 (33)	17.9 (12)
The information was presented in a way I could clearly understand	0	0	3.0 (2)	13.4 (9)	50.7 (34)	32.8 (22)
My understanding of interprofessional practice has improved as a result of having participated in this module	0	6.0 (4)	1.5 (1)	17.9 (12)	49.3 (33)	25.4 (17)
I will apply information that I learned from this module as I continue my education	0	4.5 (3)	4.5 (3)	19.4 (13)	46.3 (31)	25.4 (17)
I was satisfied with this module overall	0	4.5 (3)	1.5 (1)	28.4 (19)	37.3 (25)	28.4 (19)
I am likely to recommend this module to another student^*^	1.5 (1)	6.1 (4)	15.2 (10)	27.3 (18)	34.8 (23)	15.2 (10)

* *n = 66*.

Student evaluations of the module content were very favorable (data not shown). Most students agreed or strongly agreed that the module met the four program objectives including (1) defining the four core competencies of interprofessional education (94% agreed or strongly agreed); (2) defining population health from a public health perspective (97% agreed or strongly agreed); (3) defining prevention from a public health perspective (97% agreed or strongly agreed); and (4) recognizing real-world applications of interprofessional practice (96% agreed or strongly agreed).

The knowledge check quiz consisted of ten questions and a score of 8 correct answers constituted a passing score (see Supplementary Materials for quiz questions). More than three-quarters of students (81%; *n* = 54) passed the quiz on the first try and the rest of the students passed with two or more attempts. There was no limit to the number of attempts allowed to pass the quiz.

## Discussion

This study represents the first online IPE intervention at this school focused on introducing to undergraduate public health students the concept IPE in practice, and specifically, how public health practitioners contribute to IPE teams. This module is unique in emphasizing a non-clinical health science discipline in an IPE learning experience. Our findings indicated a high level of interest in IPE among public health undergraduates and some understanding of the value of interprofessional practice among this student population at baseline; this understanding increased after exposure to the training. In some cases, the increase was modest, given the high level of agreement prior to the training. This finding is consistent with studies of health professions students that show positive attitudes toward IPE prior to training (17). That attitude can diminish throughout their educational program if IPE is not further cultivated within the students (17).

Overall, the module was evaluated positively by learners, with two-thirds of completers reporting satisfaction with the module and 75% reporting a better understanding of interprofessional practice. These findings are promising as this is a learning opportunity intended to provide an initial exposure to IPE, and specifically, the role that public health practitioners will have in interprofessional practice. Among professionals, trends in the field—such as Public Health 3.0—suggest the increasing importance of cross-sector partnerships in affecting change in health outcomes (18). Working together across sectors can take many forms, whether it is the integration of primary care and public health, community engagement and coalition building, or other large and small efforts that ultimately require interpersonal skills (19, 20).

Curricula in health professional degree programs are becoming more prescribed to meet changing accreditation standards and needs from the field. For instance, the Council on Education for Public Health states that undergraduate public health students are expected to be trained in several related domains and concepts such as “teamwork and leadership,” as well as characteristics of health care and public health and the “influences and responsibilities of the different agencies and branches of government,” etc. (21). In addition, Master of Public Health students must be able to “perform effectively on interprofessional teams” by the end of their two-year graduate education. Given the overlap of training needs across professional and student audiences, a short 2-hour IPE module can be effective as part of a course given as an assignment or through extracurricular offerings.

### Limitations

Although the content in this module is ultimately intended for learners across multiple health professional disciplines, the module was piloted with representative learners from only one discipline, public health. The intent in piloting this module within public health undergraduate students was to understand if the content supported knowledge acquisition of IPE and public health's role within it. The durability of learning from a single module remains unclear. Further, students self-selected into the intervention; taking the training module was encouraged though not required for the undergraduate students. We do not know if the students who elected to participate have different knowledge or familiarity with IPE compared to those who did not participate. As noted in the results, some student pre-test responses were very high for some questions before the intervention, which could either indicate prior knowledge of IPE or false perceptions of IPE knowledge and skills before the intervention. Items for the survey instrument itself were drawn from other surveys. Psychometric properties for this instrument are not available. It is possible that response bias may be a factor in the students' responses, although we do not believe that is a substantive factor in our findings.

### Future Research

Future offerings of this module will extend beyond public health students to include learners from other health professions, to determine whether similar attitudes and satisfaction with the module are evident. The evaluation of future offerings will also indicate the validity of the survey instrument used to gather post-module data. In addition, because the durability of learning from a single module is unclear, the longer-term impact of integrating IPE content into courses should be explored to determine whether exposure to foundational IPE concepts leads to more effective interprofessional practice as public health professionals.

The evaluation of this module was mainly to determine learners' attitudes, satisfaction, and perceptions toward the module and its content. While there are increasing number of IPE learning experiences across health professional students at both the undergraduate and graduate level, there is still a lack of retention of behavior and skills beyond initial IPE experiences (22). Research initiatives are currently in place at our institution to examine the application of IPE experiences within the context of professional practice of recent health professional graduates from at least four disciplines including public health.

## Data Availability

The datasets generated for this study are available on request to the corresponding author.

## Author Contributions

OA (with others) developed and implemented the IPE module, generated the idea for the manuscript, co-developed the pre- and post-test evaluations, and drafted the introduction section and abstract. EA interpreted the results, drafted the results section and edited the final version of the manuscript. PG (with others) developed and implemented the IPE module and accompanying materials, co-developed the post-test evaluation and quiz, drafted the methods section, and assembled the appendix. EY conducted the data analysis and oversaw student recruitment. AB drafted the discussion section and implemented critical revisions to the manuscript. All authors contributed to drafting and revisions, approved the final version for publication, and agreed to be accountable for all aspects of the work in ensuring that questions related to the accuracy or integrity of any part of the work are appropriately investigated and resolved.

### Conflict of Interest Statement

The authors declare that the research was conducted in the absence of any commercial or financial relationships that could be construed as a potential conflict of interest.
