# Supplementary material for: Developing a Framework for Population Health in Interprofessional Training: An Interprofessional Education Module
Source: Front Public Health. 2019 Mar 21;7:58. doi: 10.3389/fpubh.2019.00058 (PMC6437311; doi:10.3389/fpubh.2019.00058)
Supplement: Supplementary file 1 [file Data_Sheet_1.PDF]

Supplementary Material

Developing a Framework for Population Health in Interprofessional Training: An Interprofessional Education Module

Olivia S. Anderson, Ella August*, Phoebe K. Goldberg, Emily Youatt, Angela J. Beck
*** Correspondence:** Dr. Ella August eaugust@umich.edu

Supplementary Table 1. Pre-survey questions for IPE Module *Interprofessional Practice for Population Health***.** Students were asked to indicate their level of agreement with the following statements, with response options of: strongly disagree, disagree, somewhat disagree, somewhat agree, agree, strongly agree.
● Students from different health science disciplines should be educated in the same setting to establish collaborative relationships with one another
● Care delivered by an interprofessional team will benefit the health outcomes of the patient/client
● Incorporating public health as part of an interprofessional team is crucial to address the social determinants of health for **individual** health outcomes
● Incorporating public health as part of an interprofessional team is crucial to address the social determinants of health for **population** health outcomes

Supplementary Table 2. Post-survey questions for IPE Module *Interprofessional Practice for Population Health*.
1. Please indicate your level of agreement with the following statements. Response options: strongly disagree, disagree, somewhat disagree, somewhat agree, agree, strongly agree.<br>● Students from different health science disciplines should be educated in the same setting to establish collaborative relationships with one another<br>● Care delivered by an interprofessional team will benefit the health outcomes of the patient/client<br>● Incorporating public health as part of an interprofessional team is crucial to address the social determinants of health for **individual** health outcomes<br>● Incorporating public health as part of an interprofessional team is crucial to address the social determinants of health for **population** health outcomes
2. To what extent do you agree that this training module met the following stated **program objectives**? Response options: strongly disagree, disagree, somewhat disagree, somewhat agree, agree, strongly agree.<br>● Define the four Core Competencies of Interprofessional Education<br>● Define population health from a public health perspective<br>● Define prevention from a public health perspective<br>● Recognize real-world applications of interprofessional practice
3. Please indicate your level of agreement with the following statements. Response options: strongly disagree, disagree, somewhat disagree, somewhat agree, agree, strongly agree.<br>● The presenters were effective<br>● The information was presented in a way I could clearly understand<br>● My understanding of interprofessional education has improved as a result of having participated in this module<br>● I will apply information that I learned from this module as I continue my education<br>● I was satisfied with this module overall<br>● I am likely to recommend this module to another student
4. In your estimation, approximately how much time did you spend on this module in total? Response options: less than 2 hours, 2 hours, 2.5 hours, 3 hours, more than 3 hours.

5. Did you experience a problem with the technology? Response options: yes, no. [Display logic used if response was yes to ask “Please describe the problem with the technology that you experienced.]
6. What was the most valuable part of this module?
7. Please provide comments on the delivery of the module, usefulness of the material, or any other module-related feedback.

Supplementary Table 3. Post-survey quiz questions for IPE Module *Interprofessional Practice for Population Health***.**
1. Which of the following is the definition of the values/ethics for interprofessional practice competency?<br>● Apply relationship-building values and the principles of team dynamics<br>● Share acknowledgement of each participating team member's roles and abilities<br>● Work together with mutual respect and shared values<br>● Communicate in a responsible manner that supports a team approach
2. Which of the following is the definition of the roles/responsibilities competency?<br>● Apply relationship-building values and the principles of team dynamics<br>● Share acknowledgement of each participating team member's roles and abilities<br>● Work together with mutual respect and shared values<br>● Communicate in a responsible manner that supports a team approach
3. Which of the following is the definition of the interprofessional communication competency?<br>● Apply relationship-building values and the principles of team dynamics<br>● Share acknowledgement of each participating team member's roles and abilities<br>● Work together with mutual respect and shared values<br>● Communicate in a responsible manner that supports a team approach

4. Which of the following is the definition of the teams and teamwork competency?
- Apply relationship-building values and the principles of team dynamics
- Share acknowledgement of each participating team member's roles and abilities
- Work together with mutual respect and shared values
- Communicate in a responsible manner that supports a team approach

5. True or False: Population health from a public health perspective is the health outcomes of a group of individuals, including the distribution of such outcomes within the group.
- True
- False

6. Which of the following is an example of primary prevention?
- Flu vaccine
- Mammography
- Chemotherapy

7. Which of the following is an example of secondary prevention?
- Flu vaccine
- Mammography
- Chemotherapy

8. True or False: The Health in All Policies approach requires cross-sector collaboration.
- True
- False

9. True or False: Principles of interprofessional practice can include collaboration across jurisdictions (federal, state, local, tribal, etc.)
- True
- False

10. True or False: Strategies for successful interprofessional practice apply only to professional-professional interactions and not to professional-patient interactions.

- True
- False
